# Virtual Community Health Promotion

**Published:** 2007-06-15

**Authors:** Richard Crespo

**Affiliations:** Marshall University School of Medicine — Family and Community Health

The "wiki" and open-source phenomena are transforming the way knowledge is generated and shared around the world. The word wiki is from the Hawaiian term wiki-wiki, which means to do something quickly (1). The term became prominent because of the online encyclopedia Wikipedia (www.wikipedia.org), which now has more than 2 million articles contributed by tens of thousands of people. People in so many fields are using Internet communities that the term *wiki* has come to refer to an online group that collectively works on a project. In the business world, the wiki concept is called wikinomics (1). Even U.S. spy agencies are using a wiki-like online community, Intellipedia, on which people with U.S. security clearances can update each other on security matters (2).

The term *open source* refers to software codes that are available online to anyone who wants to know how the software works. Traditionally, this kind of information was considered proprietary and kept highly secret. However, the treatment of such information is changing. Proctor & Gamble (P&G), for example, is opening up its research and development division by inviting entrepreneurs to design new products online. Today thousands of entrepreneurs work on product ideas for P&G through the Internet, and one entrepreneur recently earned a royalty of more than a half million dollars from P&G (1). In another example of online collaboration, professors from the Wharton School of Business and Massachusetts Institute of Technology (MIT) are writing a business book online, and more than 1000 people have signed up to participate (3). In addition, MIT is now providing free Internet access to all of its course material through a program called MITOpenCourseWare (http://ocw.mit.edu).

Community participation is central to all the wiki and open-source applications. Because people can participate in creating a product, whether an encyclopedia or a gadget, they feel a sense of community and a commitment to what they have created (4). For example, an online company called Threadless (www.threadless.com) prints T-shirts that contributors design online. Contributors submit their designs, which are then voted on by registered users of Threadless, and the designs with the highest scores get printed. The designers and company share the profits. Threadless now has a community of 400,000 registered users who vote on the T-shirt designs. According to the company's founder, the key to its success is that the company is a community first and a business second (5).

Social networks constitute another type of virtual community on the Internet (1). Perhaps the best known social network is MySpace (www.myspace.com), which has more than 100 million registered users from throughout the world. Nearly 85% of U.S. college students are registered either on MySpace or Facebook, a similar Internet social network (1).

An important characteristic of virtual communities is users' sense of "ownership," the sense that they create and thus "own" the content of a community Web site. Wikipedia is perhaps the best example of this sense of ownership. Although Wikipedia has many detractors, it also has fiercely loyal users who actively monitor entries in order to correct articles and keep vandals out. On average, users make changes to newly submitted Wikipedia articles within 3 minutes of their being posted (6).

What intrigues me about online communities is that the characteristics of community participation and ownership they exemplify are also fundamental to community health. Community health practioners, however, have fallen behind in applying these principles in the virtual world. In a recent report, an expert panel convened by the Centers for Disease Control and Prevention (CDC) recommended that public health officials should develop the idea of virtual community health promotion (7).

I think there are three ways that public health professionals can use the Internet to promote community health. One is to use the wiki format to create a bottom-up knowledge base of what works and how it works, a sort of "wikihealth," whereby a community of users would contribute to a body of community health knowledge and have complete access to that body of knowledge. The focus of "wikihealth" would be on community participation, with community members posting information about successful strategies that may not be well covered by professional publications. For example, "wikihealth" could fill a void in information about local efforts to overcome childhood obesity by serving as a repository of information concerning how obesity is addressed in schools, rural communities, urban communities, and ethnic communities. Entries could have links to relevant research articles.

A second way of using the Internet to promote community health is to establish a community organizer based on the MySpace model. A community would have its own Internet site to facilitate grass-roots organizing, collect ideas, and communicate events. An example of such a community organizer is a project sponsored by the Appalachian Regional Commission and CDC (the Appalachian Diabetes Control and Translation Project). In this project, I work with more than 50 diabetes coalitions in nine states. We have designed a Web site where each coalition may have its own page (as on MySpace), and we are in the process of helping coalitions put materials on their own pages.

A third way of using the Internet to promote community health is to use blogs to exchange information. Blogs (short for Web logs), on which people can post unfiltered information and opinions, are transforming the way that people talk with each other and share ideas. Blogs can stand alone or function as a part of the applications described above. An ambulance driver in London has a blog (http://randomreality.blogware.com/blog) that claims to have 1.5 million participants and to be visited by an average of 3000 people per day. The blog works because the blog's author is interesting, insightful, and a good writer. Community health practitioners could use blogs to share information about what works and what doesn't work. For example, diabetes coalitions from throughout the world could have a running dialogue about the effectiveness of various patient self-management strategies and thereby could learn from each other across geographic and cultural boundaries.

The possible uses of online communities in the field of community health are almost endless. To take advantage of these possibilities, community health practitioners need to enter the virtual world and apply the community health principles of community participation and ownership in new ways.
